# Quadrupling the N95 Supply during the COVID-19 Crisis with an Innovative 3D-Printed Mask Adaptor

**DOI:** 10.3390/healthcare8030225

**Published:** 2020-07-23

**Authors:** Annabel M. Imbrie-Moore, Matthew H. Park, Yuanjia Zhu, Michael J. Paulsen, Hanjay Wang, Y. Joseph Woo

**Affiliations:** 1 Department of Cardiothoracic Surgery, Stanford University, Stanford, CA 94305, USA; aimbrie@stanford.edu (A.M.I.-M.); mhpark@stanford.edu (M.H.P.); yuanjiaz@stanford.edu (Y.Z.); mpaulsen@stanford.edu (M.J.P.); hanjay@stanford.edu (H.W.); 2 Department of Mechanical Engineering, Stanford University, Stanford, CA 94305, USA; 3 Department of Bioengineering, Stanford University, Stanford, CA 94305, USA

**Keywords:** COVID-19, personal protective equipment, N95 masks, 3D printing

## Abstract

The need for personal protective equipment during the COVID-19 pandemic is far outstripping our ability to manufacture and distribute these supplies to hospitals. In particular, the medical N95 mask shortage is resulting in healthcare providers reusing masks or utilizing masks with filtration properties that do not meet medical N95 standards. We developed a solution for immediate use: a mask adaptor, outfitted with a quarter section of an N95 respirator that maintains the N95 seal standard, thereby quadrupling the N95 supply. A variety of designs were 3D-printed and optimized based on the following criteria: seal efficacy, filter surface area and N95 respirator multiplicity. The final design is reusable and features a 3D-printed soft silicone base as well as a rigid 3D-printed cartridge to seal one-quarter of a 3M 1860 N95 mask. Our mask passed the computerized N95 fit test for six individuals. All files are publicly available with this publication. Our design can provide immediate support for healthcare professionals in dire need of medical N95 masks by extending the current supply by a factor of four.

## 1. Introduction

In late 2019, an outbreak of severe pneumonia associated with influenza-like symptoms and an alarming mortality rate was discovered to be caused by the novel coronavirus, SARS-CoV-2 [1]. The resulting disease was termed COVID-19. Personal protective equipment (PPE), especially N95 respirators, are now in critically short supply in many parts of the world [2,3]. The pandemic has become a major healthcare burden in many countries, and innovative solutions are required to mitigate the shortage of PPE in order to minimize disease spread and loss of life [4].

Manufacturers are increasing production to meet demand for PPE but are limited by significant lead times. N95 respirators are a specialized type of fit-tested respiratory PPE designed to exclude over 95% of 0.3 µm particles [5] and are considered essential protection for healthcare workers against airborne viral particles [6]. Although there are ongoing sterilization efforts for used N95 masks, more research is needed to confirm the efficacy of sterilization without affecting the filtration quality of N95 masks [7,8]. Additionally, there is ongoing work to alleviate PPE shortage using homemade masks and 3D-printed mask adaptors [9,10]. Previous 3D-printed mask adaptors feature reusable designs with a frame to secure a filter material. However, due to the use of low-filtration efficacy materials as well as imperfect seals around the filter material and/or the face, these previous solutions do not achieve a medical grade N95 standard. We aimed to develop an immediate solution to mitigate this shortage: a 3D-printed mask adaptor, outfitted with a sectioned portion of an N95 respirator, that maintains the N95 filtration standard and thereby multiplies the available number of masks. Maintaining the N95 standard requires a novel mask adaptor design that conforms to the face and seals around each component of the mask and filter.

## 2. Materials and Methods

The mask adaptor design was developed with a Carbon M2 3D-Printer (Carbon, Redwood City, CA, USA) using biocompatible Silicone (SIL 30, Carbon, Redwood City, CA, USA) and Multipurpose Polyurethane (MPU 100, Carbon, Redwood City, CA, USA) resins. Only 1860 3M N95 masks (3M, Saint Paul, MN, USA) that could not be donated to the hospital were used for design development. At each iteration, the design was evaluated based on the following criteria: filter efficacy, filter surface area, and respirator multiplicity. In particular, our design goals were to properly seal all components to the N95 standard and to optimize filter surface area such that the mask maintains breathability while extending the multiplicative factor of a standard N95 respirator. Filter efficacy was quantitatively evaluated using a computerized N95 fit test machine (PortaCount Respirator Fit Tester, TSI Inc., Shoreview, MN, USA) that measures the concentration of particles leaking into the mask, either through the filter itself or through gaps around the mask components and in the face seal. Respirator multiplicity—calculated as the number of masks produced from a single N95 mask—was balanced with the need for sufficient filter surface area for comfortable breathability. Breathability was qualitatively evaluated as the number of hours a user could comfortably wear the mask; we qualitatively observed that with a cartridge design that enables a filter surface area of approximately one-quarter of the 1860 3M N95 mask, the breathability was such that a user could still wear the mask for a full workday.

A sufficient seal of our mask to the user’s face requires the mask to conform to a range of facial shapes, even while talking and moving. We selected the soft biocompatible silicone material for the base of the mask to perform this function. Additionally, we utilized a teardrop shape rather than the 1860 N95 dome shape to conserve material and weight. The final silicone base design was also molded as a proof-of-concept to pursue alternative large-scale manufacturing solutions. For a manufacturing alternative, we used a three-part mold design with 30A shore hardness urethane (VytaFlex 30, Smooth-On, Macungie, PA, USA). This molding alternative was a validation to confirm that our mask base geometry was compatible with traditional molding methodologies; if molding is desired for the final product, we recommend biocompatible silicones or urethanes with material properties similar to that of SIL 30 (approximately 35A shore hardness). 

A 3D-printed rigid cartridge was developed to allow for a larger filter surface area and to facilitate single-use filter loading and unloading with the cutting and sealing process of the N95 mask sections performed separately. We designed the cartridges to follow the contour of the sectioned quarters of an N95 mask. Non-toxic thermoplastic adhesive (All Purpose Glue, Arrow Fastener Co., Saddle Brook, NJ, USA) was used to seal the filter section to the cartridge on both sides. Additionally, the cartridge features a flange that fits into an undersized slot in the silicone mask base, thus sealing the cartridge to the base without the need for adhesive. 

To validate that it is possible to maintain the N95 filter efficacy standard with our novel mask adaptor, the final prototype was tested on six individuals using the PortaCount computerized N95 fit test machine. The fit test was performed by affixing a probe to the filter section of the mask through which the machine measured the concentration of particles leaking into the mask. The fit tester machine quantitatively evaluates the preservation of mask functionality, including its ability to filter particles as well as the presence of any leaks in the mask adaptor components connecting the N95 filter section to the face. For each individual, a new filter and cleaned mask was provided. Each test individual completed all four exercises: bending over, talking, and horizontal and vertical head motion.

## 3. Results

**Figure 1:**
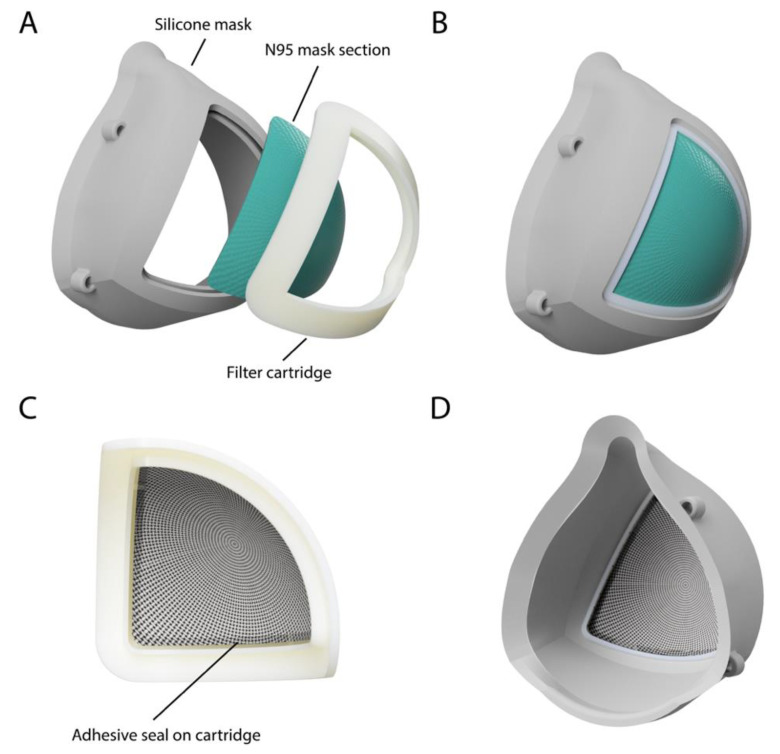
(**A**) Labeled exploded render of the final mask design with a reusable soft silicone base and a 3D-printed filter cartridge. (**B**) Front view of the final mask assembly. (**C**) The cartridge is assembled separately with one-quarter of a 3M 1860 N95 mask and adhesive to ensure a seal on the edges of the filter. (**D**) Back view of the final mask assembly.

**Figure 2:**
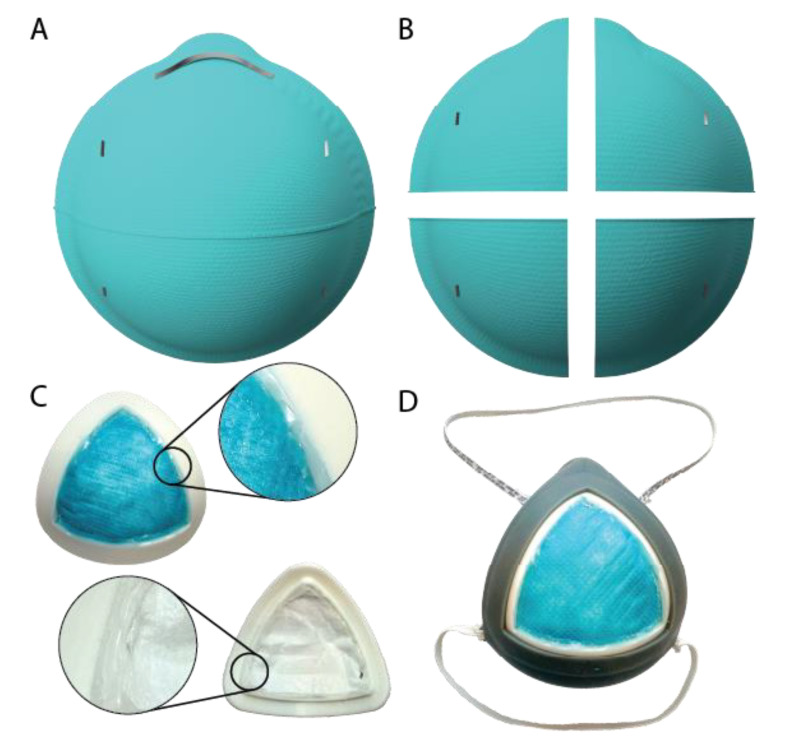
(**A**) Mask assembly begins with a single 3M 1860 N95 mask. (**B**) The metal nose strip and elastic straps are carefully removed, and the mask is cut along its horizontal and vertical axes. (**C**) Each mask quarter is placed in a cartridge face down (any asymmetries can be trimmed) and sealed to the cartridge side using non-toxic adhesive. The filter assembly is then flipped face up and adhesive is used to seal the mask section to the inner edge of the cartridge. Magnified image callouts show the adhesive seals in more detail. (**D**) Elastic straps are attached through the four fixation points and the assembled cartridge is loaded into the silicone mask base by sliding the cartridge flange into the slot in the mask base.

The final design features a rigid 3D-printed cartridge with an inner ridge used to seal one-quarter of a 3M 1860 N95 mask with non-toxic adhesive (Figure 1). Additionally, the design includes a 3D-printed soft silicone base with a slot that seals around the cartridge. The silicone base was successfully molded as a dimensionally accurate proof-of-concept mask produced using a conventional two-part molding resin. The full assembly of the mask is detailed in Figure 2. The first step is to carefully remove the metal nose strip and elastic straps from the original N95 mask, followed by cutting the mask along its horizontal and vertical axes; a 3D-printed stencil (file provided in the Supplementary Materials) can be used to assist this cutting. Each mask quarter is placed in a cartridge face down and any asymmetries can be trimmed such that the filter section sits snug within the allotted grove of the cartridge. A non-toxic thermoplastic adhesive is then applied along the entirety of the ridge, sealing all gaps between the edge of the filter and the ridge of the cartridge. The filter assembly is then flipped face up and adhesive is again used to seal the mask section to the inner edge of the cartridge for a robust seal. To assemble the reusable silicone mask base, elastic straps are attached through the four fixation points. To insert a cartridge for use, all sides of the assembled cartridge slide into the slot in the mask base. 

**Figure 3:**
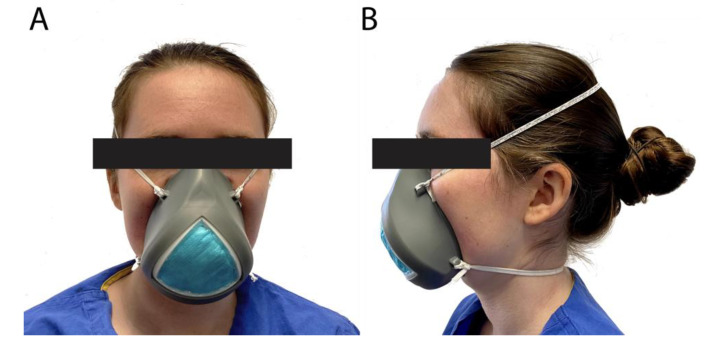
Picture showing a user wearing the final mask from the front (**A**) and side (**B**).

Figure 3 shows a user wearing the final mask. For testing, the quarter filter section of the mask was outfitted with the standard N95 fit test probe. The six individuals passed the computerized fit test wearing the mask. The PortaCount Respirator Fit Tester measures the concentration of microscopic particles outside the mask and then measures the concentration that leaks inside the mask. The ratio of these two numbers is the fit factor, which is the standard for assessing a proper mask seal and is used annually to test for N95 mask fitness for healthcare workers at our institution. The overall fit factor measured was 148 ± 29, with 100 set as the standard pass level for an 1860 N95 mask. The respective scores for bending over, talking, horizontal and vertical head movements were 154 ± 49, 132 ± 38, 161 ± 38 and 169 ± 32. Note that the scores outputted on the fit test machine as “200+” were counted as 200 for this test. 

## 4. Conclusions

Our novel 3D-printed mask adaptor and cartridge is designed to extend the supply of N95 masks in a cost-effective and scalable manner by dividing each N95 mask into four sections, thus quadrupling the supply. Filter efficacy of the mask was quantitatively evaluated using a computerized N95 fit test machine, confirming the use of this mask adaptor design as a valid means of extending the mask supply while maintaining the N95 standard. 

Each component of the mask can be produced through 3D-printing or molding modalities for large-scale production, and, if necessary, edited or scaled to help accommodate a wider range of faces. The filter cartridges can be separately assembled by adhering quartered sections of an N95 mask to the filter holder, which can then be supplied to the users. Before each high-risk exposure, the cartridges have been designed to quickly assemble with the mask base, while properly maintaining its seal. The filter sections can be peeled from the cartridge, and the cartridges and mask base can both be sanitized for reuse; note that the mask components should be discarded if any warping occurs. The exact protocol of cartridge and mask reuse should be decided upon on an institutional basis. 

Due to the immediacy of this PPE shortage, our primary goal was to rapidly develop a mask that satisfied N95 functional criteria while extending the existing mask supply. With the submission of this study, we will have included the files of our mask components so that individuals can use this work to alleviate mask shortages while making modifications to improve the fit, comfort and weight. Just as healthcare workers must go through the fit test process with standard N95 masks to find the most appropriate mask shape that can properly seal to their face, the fit test would be necessary for users to confirm proper fit with our mask. In particular, fit factors for individuals vary across different N95 respirators [11], and a follow-up study should be performed to optimize mask shape based on the fit factors measured across a random sampling of all healthcare workers. Nonetheless, by including all 3D-printing files, institutions can make adjustments for individuals to customize the mask fit. We additionally intend to adjust our design for use with the 3M 1870 N95 mask, and we will continue to explore alternative designs to improve the speed and cost of manufacturing. 

In conclusion, to alleviate PPE shortage due to COVID-19, we developed a mask adaptor system that can be outfitted with one-quarter of a section of an N95 mask while maintaining the standard level of seal. With this mask adaptor, we can effectively multiply the existing N95 supply to provide protection for frontline healthcare workers during this pandemic.

## Supplementary Materials

The following are available online at https://www.mdpi.com/2227-9032/8/3/225/s1, STL file of the mask base, STL file of the mask cartridge, STL file of the filter cutting stencil.
